# Mechanical and Thermal Influences on Microstructural and Mechanical Properties during Process-Integrated Thermomechanically Controlled Forging of Tempering Steel AISI 4140

**DOI:** 10.3390/ma13245772

**Published:** 2020-12-17

**Authors:** Bernd-Arno Behrens, Kai Brunotte, Tom Petersen, Julian Diefenbach

**Affiliations:** Institute of Forming Technology and Machines, Leibniz University Hanover, An der Universität 2, 30823 Garbsen, Germany; behrens@ifum.uni-hannover.de (B.-A.B.); brunotte@ifum.uni-hannover.de (K.B.); petersen@ifum.uni-hannover.de (T.P.)

**Keywords:** thermomechanical treatment, forging, mechanical properties, tempering steel

## Abstract

Thermomechanical treatment (TMT) describes the effect of thermal and mechanical conditions on the microstructure of materials during processing and offers possible integration in the forging process. TMT materials exhibit a fine-grained microstructure, leading to excellent mechanical properties. In this study, a two-step TMT upsetting process with intermediate cooling is used to demonstrate possibilities for a process-integrated treatment and corresponding properties. A water–air-based cooling system was designed to adjust different phase configurations by varying the target temperature and cooling rate. Four different thermal processing routes and four combinations of applied plastic strains are investigated in standardized mechanical tests and metallographic analyses. The applied TMT results in a finely structured bainitic microstructure of the investigated tempering steel AISI 4140 (42CrMo4) with different characteristics depending on the forming conditions. It can be shown that the demands of the standard (DIN EN ISO 683) in a quenched and tempered state can be fulfilled by means of appropriate forming conditions. The yield strength can be enhanced up to 1174 MPa while elongation at break is about 12.6% and absorbed impact energy reaches 58.5 J without additional heat treatment when the material is formed after rapid cooling.

## 1. Introduction

Forged components made from alloyed tempering steel are commonly used for parts with demands on safety and durability for example in the aviation industry or in automotive applications. These components must be both lightweight and highly resistant to dynamic loads [1]. During the manufacturing of such parts, the forging process is one of the major influencing factors. In addition, the parts’ properties are influenced by surrounding processes such as heat treatment and by input parameters such as material composition [2]. The combination of these factors is summarized as thermomechanical treatment (TMT) and leads to excellent properties, which could not be achieved by the individual processes if they are performed separately. The excellent strength and toughness of TMT parts results from the fine-grained microstructure [3]. In addition, this type of heat treatment enables integration into the forging process chains, which can lead to considerable savings in time and costs [4]. Nevertheless, the transfer of these technologies into broad applications in the forging industry is still pending. They are already widely used in the manufacturing of semi-finished products, for example in the sheet metal sector [5] or extrusion processes [6].

The objective of this work is to prove the possibility of replacing a conventional process setting of forging and heat treatment by process-integrated TMT. The influence of TMT on the microstructure is used to adjust component properties. The approach seeks to provide a basis on which forming processes with locally adapted properties can be developed. This will make it possible to design the forming process in such a way that particularly highly stressed areas of the component can benefit from the outstanding properties of TMT. In areas with lower loads, different properties can then be tolerated. To model a two-stage forging process with thermomechanical process control, an upsetting process consisting of primary and secondary forming with intermediate cooling is applied, as described in detail in the following material and method description. Results from a test matrix including four different thermal processing routes and four combinations of applied plastic strains were investigated.

### Process-Integrated Thermomechanical Treatment

Thermomechanical treatment processes are used to fundamentally change material structures and mechanical properties. A unique material state can be created by a targeted exploitation of mechanical effects from plastic forming and thermal influences from heat treatment. Three essential aspects must be taken into account if such a technology is to be transferred to industrial practice: material, mechanical properties, and production process.

In terms of material selection, TMT materials have gained particular importance in the production of fine-grained structural steels, which are characterized by low contents of alloying elements and a fine-grained structure. In addition to this, the precipitation of micro-alloy elements such as niobium or titanium leads to the high strength of these steel grades [7]. Aspects of the thermomechanical processing of the technically particularly relevant tempering steel AISI 4140 are known from the literature as well. According to Weise, TMT combined with forming leads to a finer structure of ferritic grains and carbide-rich phases [8]. Furthermore, Lv et al., presented mechanical properties (up to 2022 MPa yield strength) related to fine-grained microstructures produced by hot rolling and direct quenching [9]. To improve the applicability of the technology for this widely used material, further material-specific investigations are necessary.

For the determination of the mechanical properties influenced by TMT, small-scale processes with focus on the investigation of microstructures by metallographic methods were mainly used. Additionally, reduced test procedures are common for the determination of mechanical properties [10]. Kang et al., for example, examined a cold-rolled and tempered steel with an ultra-fine grain structure by means of hardness measurements and small-scale tensile tests with a cross-section of 2 × 2 mm [11]. The use of standard-compliant test specimens in regular dimensions for the evaluation of process-influenced microstructures is also known for individual configurations of processes and materials. Wen et al., for example, investigated the mechanical properties of a high-strength-low-alloy steel (HSLA) as a function of different microstructures caused by a variation of the parameters during normalization using tensile and Charpy pendulum impact tests. In this case, a reduction in the grain size by approx. 30% was accompanied by an increase in tensile strength by approx. 12% [12]. Mayo et al. investigated the tensile strength of micro-alloyed steel after TMT at different temperatures using tensile specimens with 4 mm diameter [13]. Jia et al. produced a fine-grained ferritic microstructure with spherically shaped cementite by multi-pass rolling at temperatures between 720 and 820 °C in a steel with 0.47 wt % C. The mechanical properties were investigated in tensile and impact tests and reached the values for the quenched and tempered reference material. The microstructure was described as “ferritic-pearlitic” [14]. In the field of bainitic steels, Hui et al. presented investigations on tensile specimens with a diameter of 5 mm. Depending on the temperature during forming, significant increases in strength were found [15]. To enable the use of this technology in industrial design processes, it is necessary to provide reliable values of the mechanical properties such as tensile strength and absorbed impact energy corresponding with the microstructure and the forging parameters. It was therefore crucial to carry out the corresponding standardized test procedures with macroscopic test specimens with low scaling influence.

Challenges in process control inhibit the use of this technology within the industrial production process of the hot forming of individual parts, although its importance is already well-known in science. For example, the basic influence represented by the transformation behavior of the material during process-integrated TMT is currently under investigation [16]. Additionally, influencing factors, measuring methods and simulation possibilities were discussed by Tekkaya et al. [2]. A significant impact results from the type of microstructure during forming. This depends above all on the forming temperature and the cooling rate which is applied to approach it from the austenitizing temperature [17]. The integration of TMT process steps into forging processes has great potential but requires further research.

## 2. Materials and Methods 

The tempering steel AISI 4140 was chosen for the examination due to its widespread use in the industrial production of safety-relevant components. The analysis of the chemical composition of the initial material by spark spectrometry shows a good agreement with the contents of alloying elements specified in the corresponding standard (DIN EN ISO 683) [18]. A selection of the most important elements of the chemical composition is given in Table 1.

A two-step process is presented, consisting of a primary forming operation and secondary forming. The primary forming is carried out at the usual hot-forging temperature (1200 °C) to simulate the forming of the part’s shape. This is necessary because the secondary forming, which is used as a thermomechanical treatment, must be carried out on a significantly lower temperature level. The reduced forming temperature (800 °C or less) results in a reduced total formability and increases the required forming force. Higher temperatures would inhibit the formation of the fine-grained target structure with desired properties, as these would promote recrystallization during forming. Forming leads to an increase in dislocation density in the material, which represents an energy storage. If this energy is still sufficiently available at the time of transformation of austenite into the low temperature modification, it is dissipated by the formation of new grain boundaries. The cooling rate and the temperature during secondary forming are particularly important influencing parameters. Rapid cooling is required to reduce the loss of “stored” forming energy. Additionally, the low-temperature modification the material transforms to from austenite depends on the cooling rate. Slow cooling results in ferritic-perlitic microstructure whereas very rapid cooling leads to martensite. In addition, a bainitic microstructure can be achieved with controlled cooling, the properties of which can be classified between ductile ferrite-pearlite and hard martensite.

**Figure 1:**
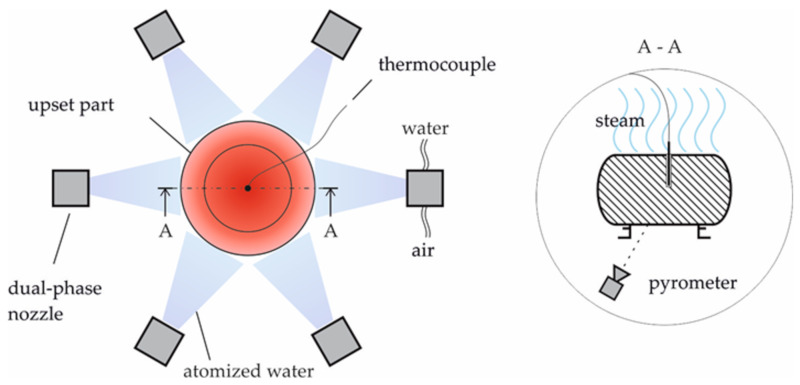
Scheme of the cooling station: top view (**left**) and sectional view (**right**).

To ensure this controlled cooling, a cooling device allowing for temperature adjustment by applying a predefined cooling regime was designed as shown in Figure 1. Six radially arranged spray nozzles are pointed at a part that is to be cooled. The nozzles atomize water with compressed air. The temperature is recorded by means of thermocouples in the part’s center and a pyrometer facing the surface of the bottom. In this zone, the measurement of the pyrometer is only minimally disturbed by rising steam or the atomized water. To adjust different cooling rates, the flow rate and the pressure of water and air can be set. As it is not possible to record the core temperature during the forming process, a correlation between core and surface temperature was established in preliminary tests. The cooling process is stopped automatically when a defined temperature is reached. During the preliminary tests, the transformation behavior of the material during cooling was investigated, and suitable shut-off points corresponding to specific temperatures for secondary forming were derived from this. 

**Figure 2:**
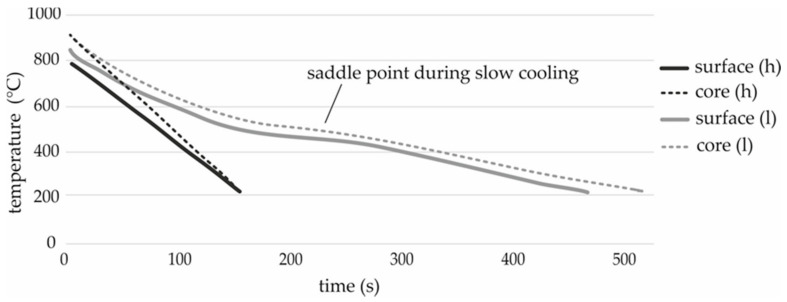
Temperature recorded during cooling with high cooling rate (black) and low cooling rate (gray).

Depending on the cooling rate, the austenitic microstructure changes in different low-temperature modifications during cooling. A low cooling rate results in a diffusion-controlled transformation and a ferritic-perlitic microstructure whilst a very high cooling rate leads to a diffusion-free transformation and a martensitic microstructure. The bainitic transformation is an intermediate stage in which, depending on factors such as the cooling rate, different transformation mechanisms lead to different forms of the bainitic structure. Figure 2 shows temperature records during the cooling of test components. The material transforms into a predominantly bainitic microstructure at both cooling rates. At the lower cooling rate (l), the diffusion-controlled transformation, indicated by a saddle point, increases in importance. In contrast to the diffusion-free transformation, the exothermic reaction during the diffusion-controlled transformation counteracts cooling. In addition, the perlitic portion of the microstructure increases.

The core temperature was used to define four different thermal process routes corresponding to different phase conditions in the material as given in Table 2. In the hh and lh routes, forming is carried out at 610 °C (comparatively high temperature) and in the hl and ll routes at 460 °C (comparatively low temperature). The high temperature is slightly above the beginning of the mentioned exothermic transformation. The material conditions of the low secondary forming temperature depend on the cooling rate (high cooling rate: h, low cooling rate: l). This rate is exposed to temperature-dependent changes in the heat transfer behavior and is affected by the exothermic transformations which lead to strong variations. For this reason, the cooling rate can only be given approximately for the relevant temperature range of 900–650 °C (before the transformation starts). The lower cooling rate (2.5 Ks^−1^) allows the material to transform in a partly diffusion-controlled manner from austenite to a bainitic structure with low pearlite content. In the case of the ll route, the transformation is finished before secondary forming is applied. The approach pursued by the hl route is to suppress the diffusion-controlled transformation by means of a higher cooling rate (5 Ks^−1^). The material thus transforms to lower bainite during forming without additional pearlite.

**Figure 3:**
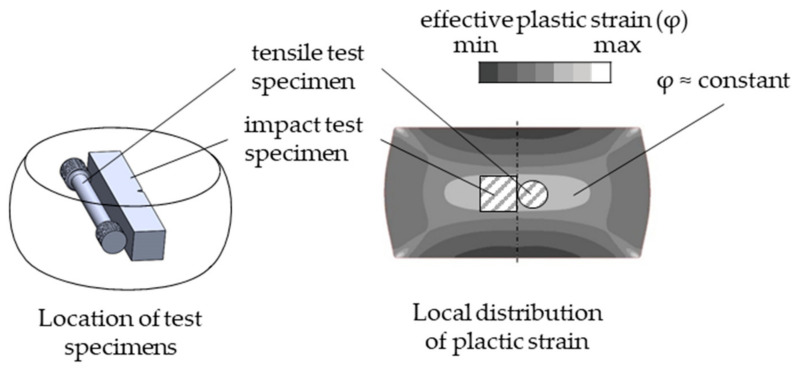
Formed component with distribution of plastic strain (schematically) and position of test specimen.

In order to provide specimens for standardized tests, such as Charpy pendulum impact tests (DIN EN ISO 148 [19]) and tensile tests (DIN EN ISO 6892 [20]) with regular sized specimens according to international standards, the forgings had to have an area of approximately homogeneous deformation large enough to extract them. In this way, a correlation of the thermal and mechanical forming conditions with the resulting properties can be derived. Thus, a cylindrical upsetting geometry with initial dimensions of Ø 50 mm × 80 mm was chosen. Figure 3 schematically shows the distribution of plastic strain and the position of the test pieces cut out by wire EDM. 

The microstructure was examined by means of metallographic cuts. To ensure the most direct assignment between microstructure and mechanical properties, a specimen is machined from each component for each test procedure. Beside the tensile (round) and impact test specimen (square), a small additional specimen was also cut out in between. These were ground, polished, and etched with nitric acid for metallographic examination. For each parameter combination, three components were processed into test specimens. Round tensile test specimens (form B) with a diameter (*d*_0_) of 5 mm and a gauge length (*L*_0_) of 25 mm (DIN 50,125 [21]) were manufactured and tested according to the standard (testing speed according to method A) [20]. In accordance with the standard, specimens for Charpy pendulum impact tests with V-notch and a cross section of 10 mm × 10 mm and a length of 55 mm were prepared [19]. All mechanical tests were carried out at ambient conditions.

In order to standardize the microstructure of the blanks across the cross section and to exclude effects from the production of semi-finished products, the blanks were fully annealed before the process. The part temperature was only recorded during cooling in the cooling station. The raw part was heated and primary formed by upsetting at a hot forging temperature of 1200 °C. All forming operations were carried out on a hydraulic press (Schirmer & Plate) with a ram speed of 30 mms^−1^. Afterwards, the secondary forming temperature was set using the cooling station and the part was formed to its final height. Subsequently, the parts were cooled in an uncontrolled manner at ambient atmosphere. Reference parts (ref) were forged in a single step to the final height at hot forging temperature.

To determine the influence of mechanical deformation during forging, four combinations of the applied effective plastic strain (*φ*) in primary and secondary forming were investigated and are presented in this paper. The relevant central part area (see Figure 3) is subjected to two different levels of effective plastic strain during primary forming: *φ*_1_ = 0.3 (height 62 mm) and *φ*_1_ = 0.6 (height 51 mm). The effect of *φ*_1_ was expected to be minor, and therefore only one set of parameters containing *φ*_1_ = 0.6 was investigated. Subsequently, the part was upset in secondary forming with effective plastic strains of *φ*_2_ = 0.3, *φ*_2_ = 0.6 and *φ*_2_ = 0.9 representing the thermomechanical treatment. As listed in Table 3, the denomination of these mechanical processing routes corresponds to the applied plastic strain.

## 3. Results

The description of the metallographic analyses focuses on the influence of the mechanical processing route since a detailed discussion of the impact of the thermal regime was already given in a previous publication [22]. The resulting mechanical properties are clearly stated in summarizing diagrams for both parameters, so this paper gives a complete overview of the results of the entire test matrix.

### 3.1. Metallographic Examination 

**Figure 4:**
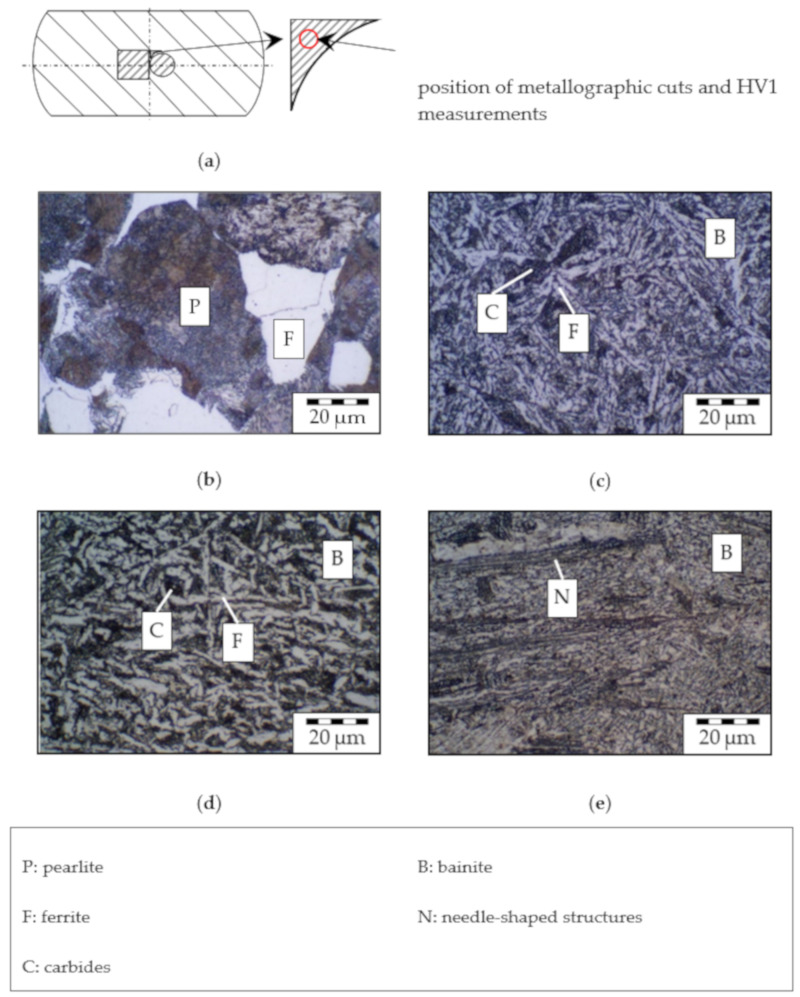
(**a**) Position of metallographic cuts and HV1 measurements, micrographs: (**b**) initial state [22]; (**c**) reference route and different thermomechanical treatment (TMT) routes ((**d**) 0.3 + 0.3, lh; (**e**) 0.3 + 0.3, hl). Etched with nitric acid (5%).

The micrographs in Figure 4 show that the structure of the initial state consisting of ferrite (F) and pearlite (P) (b) is primarily transformed into bainite (B) consisting of ferrite (light) and carbides (C) such as cementite (dark) after forging and TMT. Additionally, all process routes, even the reference (c), lead to significant grain refinement compared to the initial state, although this random distribution structure does not allow for a quantitative determination of the grain size.

Regardless of the mechanical route, all microstructures of parts processed in the lh route show structures similar to the example given in (d) consisting of upper bainite comparable to the reference. In this case, different mechanical routes result in a reduction in the size of the structures with increasing deformation during secondary forming. In contrast, the thermal process route with rapid cooling to a low temperature level (hh) results in a microstructure classified as lower bainite with a characteristic needle-shaped structure (N) (e).

**Figure 5:**
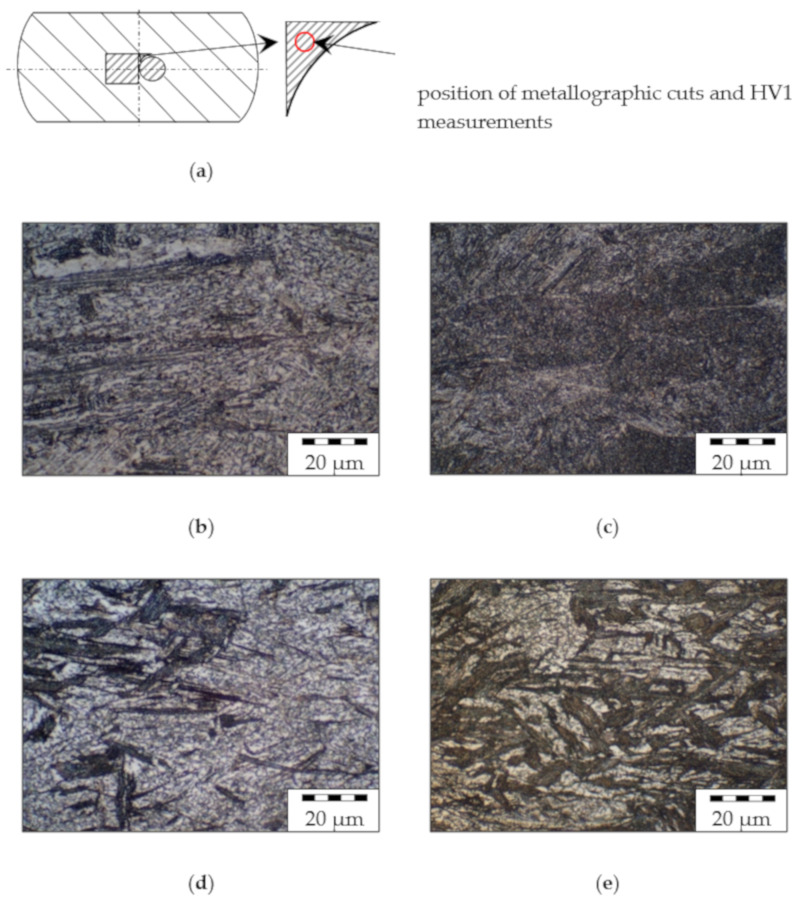
(**a**) Position of metallographic cuts and HV1 measurements, micrographs from TMT routes with high cooling rate and low secondary forming temperature (hl): (**b**) 0.3 + 0.3; (**c**) 0.6 + 0.3; (**d**) 0.3 + 0.6; (**e**) 0.3 + 0.9. Etched with nitric acid (5%).

As depicted in Figure 5, an increase in effective plastic strain leads to increasing content of these needle-like structures. Parts with minimum deformation (*φ*_2_ = 0.3) in secondary forming (Figure 5b,c) show the beginning of the formation of this phase in a fine-grained ferritic matrix. In Figure 5d, the content is elevated, corresponding to the medium effective plastic strain (*φ*_2_ = 0.6). For the maximum deformation (*φ*_2_ = 0.9), the clearest expression of this phase was observed as depicted in Figure 5e.

### 3.2. Mechanical Properties

**Figure 6:**
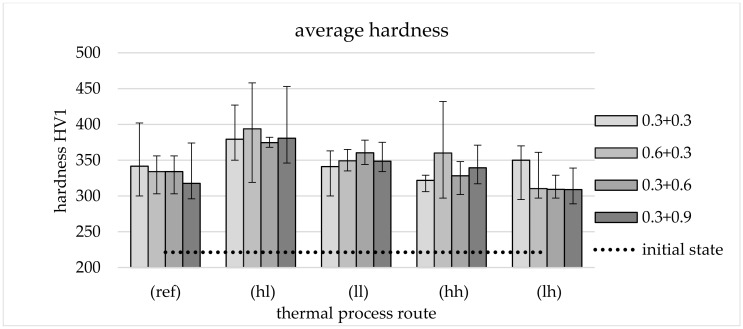
Hardness HV1: initial state, reference and different TMT routes with range of values.

The hardness of the specimens was measured according to the Vickers method (HV1) in metallographic examinations. Figure 6 shows that forming always leads to hardening compared to the initial state with the greatest effect for the hl route. With the exception of the 0.3 + 0.3 route, all mechanical processing variants show the lowest averaged hardness for the thermal lh route. Here, the measured values are already below those of the reference route. When processing along the ll and hh routes, the level of the reference is roughly maintained. Figure 6 gives the average values from three measuring points of three samples per supporting point.

#### 3.2.1. Tensile Strength

**Figure 7:**
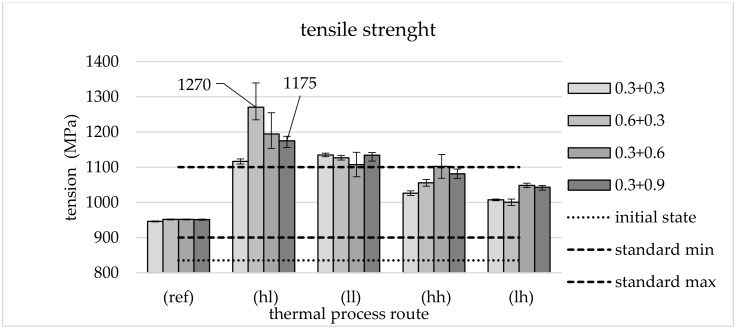
Tensile strength: Initial state, reference, different TMT routes and requirement of the standard, average of three tests and range of values.

Compared to the reference, processing on all thermomechanical routes leads to improved tensile strength, as shown in Figure 7. It is even clearer that all forming variants, including those of the reference route, lead to a significant increase in tensile strength compared to the semi-finished product. As expected, the increase is most significant for processing along the hl route. Here, too, the influence of the different mechanical routes is most clearly visible. As the effective plastic strain decreases in secondary forming, the increase in tensile strength becomes stronger, whereas the 0.3 + 0.3 route does not fit into this trend. The highest strength of 1270 MPa was determined on components from the mechanical 0.6 + 0.3 route. The maximum *φ*_2_ leads to an average tensile strength of 1174 MPa. 

In the other thermal routes, this mechanical combination does not lead to above-average increases in tensile strength. The increase in the lh route is the smallest, irrespective of the applied plastic strain. 

The different effective plastic strains in the first forming (*φ*_1_) of the two routes with *φ*_2_ = 0.3 in secondary forming (0.3 + 0.3 and 0.6 + 0.3) only affect the thermal hl route. The lowest measured tensile strength is shown here for the route with *φ*_1_ = 0.3 and the highest is for *φ*_1_ = 0.6. In the other thermal routes, no significant influence of *φ*_1_ can be seen.

#### 3.2.2. Elongation at Break

**Figure 8:**
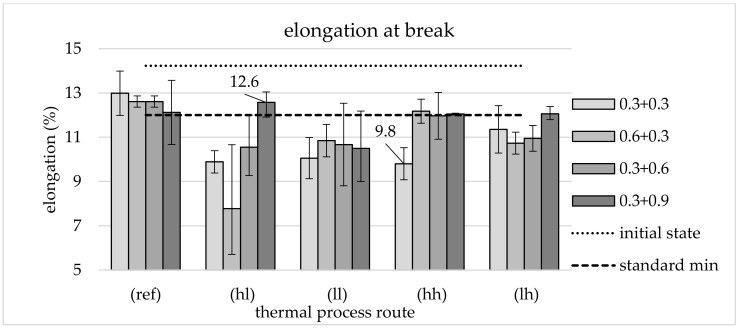
Elongation at break: initial state, reference, different TMT routes and requirement of the standard, average of three tests and range of values.

The development of the elongation at break dependent on the forming conditions is shown in Figure 8. In contrast to the tensile strength, a negative effect of the thermomechanical routes compared to the reference can be observed here. In addition, the elongation at break of all processing variants is clearly below that of the semi-finished product.

In the case of the hl route, the influence of the different mechanical routes is also clearest here. In contrast to the decrease in elongation at break for the route 0.6 + 0.3, an increase (to 12.6%) can even be achieved by processing on the route with the greatest deformation in the secondary forming process (0.3 + 0.9).

The values determined for the mechanical process routes under the other thermal conditions are much closer together. Both routes with slow cooling (ll and lh) mostly provide values for the elongation at break of about 11%. An exception is the mechanical route 0.3 + 0.9. Components from this route tend to show higher values, as with the case of slow cooling. 

For processing along the hh route, values of approx. 12% are recorded for most mechanical routes, which is slightly below the reference. The mechanical route 0.3 + 0.3 is excluded. With 9.8%, it shows a significantly lower value. In the other thermal processing routes, no anomalies like this were observed for this mechanical route with respect to elongation at break.

#### 3.2.3. Impact Energy

**Figure 9:**
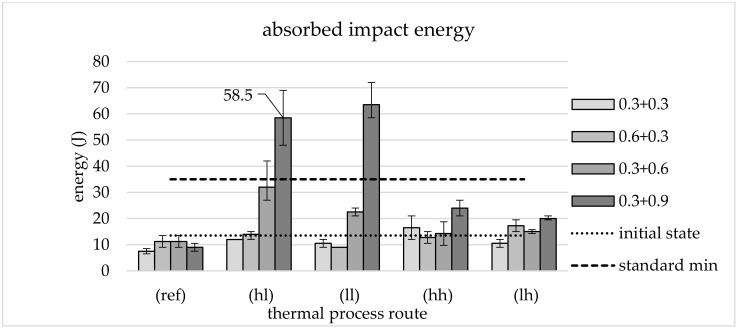
Absorbed impact energy: Initial state, reference, different TMT routes and requirement of the standard, average of three tests and range of values.

This characteristic value accordingly shows the greatest differences in the dependence on both thermal and mechanical processing parameters as shown in Figure 9. With the exception of processing at low temperature after slow cooling with low *φ*_2_ (ll, 0.6 + 0.3 and 0.3 + 0.3), all thermomechanical processing routes lead to increased values of impact energy compared to the reference route. 

Concerning the hl route, an increasing deformation in the secondary forming process to *φ*_2_ = 0.6 and *φ*_2_ = 0.9 leads to considerable improvements. Average impact energy values of 32 J for *φ*_2_ = 0.6 were determined. An increase to 58.5 J was measured for the maximum applied effective plastic strain. This maximum *φ*_2_ also shows the maximum impact energy of 63.5 J for the thermal ll route.

Values of 13.5 J were measured for the initial state. Both routes with a low degree of deformation in the secondary forming stage remain at this level, irrespective of thermal processing. All components from the mechanical reference route show even lower impact energy. 

The thermal routes hh and lh differ only slightly from each other and from the semi-finished product. The effective plastic strain in the primary forming φ_1_ does not seem to have any influence on the impact energy, regardless of thermal processing.

## 4. Discussion

The investigations on different thermal and mechanical process parameters during the process-integrated TMT of tempering steel AISI 4140 proved the significant influence of this processing on the microstructure and the mechanical properties of components. Bainitic microstructures with different characteristics were created depending on temperature during forming, cooling rate, and applied plastic strain.

When considering the hardness values, it is noticeable that the columns in Figure 6 for the different effective plastic strains in primary and secondary forming are relatively close to each other and show similar trends for the different thermal routes. This is interpreted to mean that the influence of the applied effective plastic strain (within the test matrix examined) on the hardness of the samples is rather small. A possible hardening that could result from secondary forming would cause an increase in hardness as the applied effective plastic strain increases. This is not detectable in the hardness measurements.

With regard to strength properties, TMT forming leads to high values partly exceeding the interval defined by the standard (DIN EN ISO 683) of 900–1100 MPa, which can be used to adjust ductility properties by an adapted selection of TMT parameters such as forming temperature. The very high strength of components from the mechanical 0.6 + 0.3 route is particularly striking. The high strength of 1270 MPa fits the remarkably low proportion of (white) ferritic phases in the micrographs (Figure 5c). Furthermore, it is clear that not all thermal routes are suitable for setting the target microstructure and thus achieving the high strength values. Especially with lh cooling, it can be seen that the low degree of deformation in the secondary forming process leads to the formation of a microstructure (Figure 4d) resembling that of the reference (Figure 4c). It is also remarkable that the tensile strength of components from thermal routes ll, hh, and lh seems to be largely independent of the applied effective plastic strains *φ*_1_ and *φ*_2_ (within the framework of the test matrix).

Ductility was investigated based on elongation at break and impact energy, with elongation at break in particular being identified as a critical parameter. The significant decrease in elongation at break for the route 0.6 + 0.3 is particularly remarkable. As with the tensile strength, a parallel is drawn here with the low proportion of ferritic phases. In contrast to routes with secondary forming with *φ*_2_ = 0.3 and *φ*_2_ = 0.6, the default value of 12% can be reached by the lh route with *φ*_2_ = 0.9. This can be explained by the finer structures created during the processing on this route. If rapid cooling (cooling rate 5 Ks^−1^) to a low temperature level (460 °C) (hl) is used, sufficient values for this parameter can be achieved and absorbed impact energy can be increased significantly. An increase to 58.5 J was measured for the maximum applied effective plastic strain, which clearly meets the standard’s specifications of 35 J. This maximum *φ*_2_ is also required for thermal route ll in order to achieve a sufficiently high impact energy, which reaches 63.5 J. This represents the highest averaged impact energy within the study. It is clear that a minimum plastic strain is required in order to transfer the material to a state that allows a significant increase in impact energy. This minimum is not reached for *φ*_2_ = 0.3. Processing with the 0.3 + 0.6 route leads to a significant, albeit insufficient, increase. As a minimum, *φ*_2_ = 0.9 seems to be necessary to reach sufficient ductility properties. It is equally clear that the mechanical component of the thermomechanical treatment described by the applied plastic strain *φ*_2_ is not the only influential factor, since the increases only occur during processing at the lower secondary forming temperature. At maximum *φ*_2_, an increased level of impact energy is maintained even at higher temperatures, but this already falls well below the required mark of 35 J.

The analysis of micrographs supports the evaluation of the mechanical properties. For the routes with particularly good properties, a finely structured microstructure of lower bainite was identified, which is known for its excellent properties.

## 5. Conclusions

It can be concluded that material properties in a quenched and tempered state can be achieved without additional heat treatment by suitably selecting parameters of a process-integrated thermomechanical treatment during forging. To achieve this, however, a comparatively high degree of forming is required. In the series of tests described, only the processing with the highest applied plastic strain of *φ*_2_ = 0.9 could reliably achieve this. This route shows sufficient average values for a tensile strength of 1174 MPa, elongation at break of 12.6% and an impact energy of 58.5 J. It should be noted that this only applies locally, where the corresponding thermal and mechanical requirements are met. In order to transfer these local properties to a larger component with areas of different thermal and mechanical influences, knowledge of the distribution of effective plastic strain and temperature during forming is necessary. A numerical simulation of the forming can provide this. These basic investigations are aimed at enabling the locally load-adapted design of forged components and processes. Companies of the forging industry could use these findings in order to offer their customers cost- and resource-efficient lightweight components. This contributes to increasing the competitiveness of lightweight construction solutions, which in turn contribute to the conservation of resources in the life cycle.

## Figures and Tables

**Table 1:** Chemical composition of tested AISI 4140 material determined by spark spectrometry (selected elements) average of five tests and range of values.

	Fe in wt %	C in wt %	Si in wt %	S in wt %	Mo in wt %	Mn in wt %	Cr in wt %	Ni in wt %
AISI 4140	96.88	0.428	0.241	0.014	0.228	0.757	1.080	0.074
	−0.04	−0.01	−0.006	−0.001	−0.002	−0.003	−0.01	−0.0
	+0.02	+0.006	+0.004	+0.001	+0.004	+0.011	+0.0	+0.001

**Table 2:** Thermal processing routes.

Name		Primary Forming	Intermediate Cooling	Secondary Forming
		Temperature in °C	Cooling Rate in Ks^−1^	Temperature in °C
hl	high cooling rate low temperature	1250	5	460
hh	high cooling rate high temperature	1250	5	610
ll	low cooling rate low temperature	1250	2.5	460
lh	low cooling rate high temperature	1250	2.5	610
ref	reference	forming in one step at 1250 °C		

**Table 3:** Mechanical processing routes.

Name	Primary Forming		Secondary Forming	
	*φ*_1_	Height *h*_1_ in mm	*φ*_2_	Final Height *h*_2_ in mm
0.3 + 0.3	0.3	62	0.3	51
0.3 + 0.6	0.3	62	0.6	43
0.3 + 0.9	0.3	62	0.9	36
0.6 + 0.3	0.6	51	0.3	43
